# Effects of aromatherapy on sleep disorders: A protocol for systematic review and meta-analysis

**DOI:** 10.1097/MD.0000000000025727

**Published:** 2021-04-30

**Authors:** Xin Song, Jiahua Peng, Weiyu Jiang, Minghua Ye, Lisheng Jiang

**Affiliations:** a College of Traditional Chinese Medicine, Jiangxi University of Traditional Chinese Medicine; b Institute of Obstetrics and Gynecology of Traditional Chinese Medicine, Jiangxi University of Traditional Chinese Medicine Nanchang, Jiangxi Province; c National College of Beijing University of Traditional Chinese Medicine, Beijing; d Institute of Health Preservation, Jiangxi University of Traditional Chinese Medicine Nanchang 330006, Jiangxi Province, China.

**Keywords:** aromatherapy, protocol, sleep disorders, systematic review

## Abstract

**Background:**

The progress of modern society and changes in lifestyle have not only increased the proportion of sub-healthy people, but also caused many people to suffer from sleep disorders and mental anxiety. Long-term lack of high-quality sleep will not only cause psychological problems such as anxiety and fatigue, but also cause physical abnormalities, such as abnormal hormone secretion, weakened immunity, neuroendocrine disorders, and high blood pressure. Therefore, the purpose of this study is to systematically evaluate the effectiveness of aromatherapy in improving sleep quality in people with sleep disorders.

**Methods:**

Computer search CNKI, SinoMed, Wanfang, PubMed, Web of science, and EMbase collect randomized controlled trials on aromatherapy to improve sleep quality of people with sleep disorders. The search time limit is to build the database until April 5, 2021. RevMan5.3 software is used for meta-analysis.

**Results:**

This systematic review will provide an assessment of the current state of sleep disorders, aiming to assess the efficacy of aromatherapy for patients with sleep disorders.

**Conclusion:**

This systematic review will provide a credible evidence-based for the clinical treatment of sleep disorders with aromatherapy.

## 1 Introduction

Sleep disorders are a serious health and public health problem in today's society, mainly manifested as insomnia, sleep-related breathing disorders, excessive sleepiness and parasomnias, and son on,^[1]^ which seriously affects people's daily life, work and physical and mental health, and is closely related to cardiovascular and cerebrovascular diseases, malignant tumors, and psychological diseases.^[2]^ According to clinical statistics, about 10% to 20% of adolescents and adults suffer from insomnia, and the incidence of sleep disorders in the elderly is 30% to 40%. Nearly 90% of the elderly experience sleep problems such as difficulty falling asleep, waking up early or increasing the number of awakenings, etc.^[3]^ Although insomnia is not a critical illness, long-term insomnia not only seriously affects people's work and quality of life, it is also an important risk factor for mental and psychological diseases, cardiovascular diseases, and diabetes, which increases the medical burden of society.^[4,5]^ Therefore, it is necessary to actively pay attention to sleep problems and seek practical and effective treatments. At present, the clinical treatment methods for sleep disorders are mainly drugs, behavioral cognitive therapy, and traditional Chinese medicine.^[6]^ In the pharmacological treatment of insomnia, the guide recommends the first choice of short and medium-acting benzodiazepine receptor agonists or melatonin receptor agonists, as well as antidepressants with sedative effects.^[7]^ However, it generally suffers from adverse reactions such as addiction, cognitive and mental impairment, respiratory depression, affecting the quality of daytime awakening, and so on, and it is resistant to drugs and easily causes rebound.

Aromatherapy is a complementary and alternative therapy that has emerged in recent years, which is defined as the application of essential oils or herbal essences extracted from natural plants via a number of delivery modes (eg, inhalation, massage, compress, whole-body or foot bath, external or internal skin absorption and ingestion) for medical objectives through odours produced.^[8]^ Aromatherapy is a kind of essential oil therapy that directly uses plant spices or indirectly extracts essence from spices. It was first used in the beauty industry and then gradually expanded to the medical field. It is mainly used to prevent, relieve, or treat certain diseases. To date, >40 plant derivatives have been identified for aromatherapy, with lavender oil, rose oil, and citrus species oils appearing to be more commonly used. Notably, aromatherapy offers a great number of benefits over other complementary and alternative medicines, as it is easy to deploy and does not require active patient involvement, special equipment, and skill set expertise or licensed experts; hence, it has become the second most common method of complementary and alternative medicines accepted by nurses.^[9]^

Therefore, this study extensively collected randomized controlled trials on aromatherapy in the treatment of sleep disorders, and used meta-analysis for data combined analysis, aiming to evaluate the efficacy of aromatherapy for sleep disorders.

## 2 Protocol registration

This systematic review protocol will be reported strictly adherence to the Preferred Reporting Items for Systematic Review and Meta-analysis Protocols.^[10]^ The protocol of the systematic review has been registered in the INPLASY website (registration number is INPLASY202140035). If there are any adjustments during the entire study period, we will fix and update the detailed information in the final report in time.

## 3 Methods

### 3.1 Literature source

#### 3.1.1 Inclusion criteria

1. Study type: The type of literature research is randomized controlled trials (RCTs).
2. Study participants: Patients with sleep disorders, sleep disorders assessed by tools (Pittsburgh sleep quality index [PSQI], SRSS, ICSD, among others), age ≥18 years, there are no limits to research subjects age, sex, race, condition duration, or intensity. Participants with serious underlying diseases, pregnant, or lactating women will be excluded.
3. Interventions: Experimental group: only use aromatherapy; control group: given routine care, placebo, blank control.
4. Outcome indicators: First, PSQI: PSQI is used to assess the sleep quality of subjects in the last month. It consists of 19 self-evaluated items and 5 other-evaluated items, of which the 19th self-evaluated item and 5 other-evaluated items do not participate in scoring. Eighteen items constitute 7 components, and each component is scored on a scale of 0 to 3. The cumulative score of each component is the PSQI total score, the total score range is 0∼2l, the higher the score, the worse the sleep quality.^[11]^ Second, SRSS Scale: There are 10 items in SRSS, and each item is divided into 5 grades (1∼5). The higher the score, the more serious the sleep problem. The lowest score of this scale is 10 (basically no sleep problems), and the highest score is 50 (most serious). Third, Clinical effectiveness including 4 grades of recovery, obvious effect, effective and ineffective. Effective rate = ([recovery + obvious effect + effective]/total number of cases) × 100%. Fourth is adverse reactions.

#### 3.1.2 Exclusion criteria

Exclusion criteria were: aromatherapy combined with other treatments methods; non-Chinese and English literature; the research object is animals; the literature with incomplete data, and it is impossible to extract the data; repeated publication of the literature.

### 3.2 Data sources and search strategies

Computer search databases were CNKI, SinoMed, Wanfang, VIP, PubMed, Web of science, EMbase. The search time range is from the establishment of the database to April 5, 2021. Use a combination of subject terms and free words to search. When screening articles, a comprehensive search will be carried out by keywords and full text. Search terms are: “Aromatherapy,” “Aromatic therapy,” “Aroma,” “Aromatic,” “Fragrance,” “Aromatic substances,” “Essential oil,” “Sleep,” “Insomnia,” “Sleep disorder,” “Sleep quality,” “Comorbid insomnia,” “Randomized controlled trial,” and “RCT.” The retrieval strategy is shown in Table 1.

**Table 1:** Search strategy used in PubMed database.

Number	Search items
1	Aromatherapy
2	Aromatic therapy
3	Aroma
4	Aromatic
5	Fragrance
6	Aromatic substances
7	Essential oil
8	1 or 2–7
9	Sleep
10	Insomnia
11	Sleep disorder
12	Sleep quality
13	Comorbid insomnia
14	9 or 10–13
15	Randomized controlled trial
16	RCT
17	15 or 16
18	8 and 14 and 17

### 3.3 Literature screening and data extraction

Two researchers independently screened the literature, extracted data, and cross-checked according to the pre-determined inclusion and exclusion criteria. In case of disagreement, it will be resolved through discussion or negotiation with the third researcher. First, after rechecking and excluding duplicate documents, a preliminary screening is performed by reading the title and abstract of the literature to exclude obviously irrelevant documents; further reading the full text for re-screening to determine whether to include. If necessary, contact the original research authors by phone or email to obtain information that is not yet determined but important to this research. The content of the data extraction includes: article title, first author's name, publication year, basic information of subjects, baseline conditions of disease, intervention/control measures and course of treatment, key elements of bias risk evaluation, outcome index result data, among others.

### 3.4 Risk of bias assessment

Two researchers independently evaluated the risk of bias based on the RCT bias risk assessment tool recommended by the Cochrane Handbook, and cross-checked the results. If there is any disagreement, it will be resolved through discussion or negotiation with the third researcher.^[12]^

### 3.5 Statistical analysis

RevMan5.3 software was used for meta-analysis. Mean difference was used for measurement data, odds ratio was used for classification data, and 95% confidence interval was used as the statistical analysis quantity. The heterogeneity among the included research results was analyzed by *χ*^2^ test (test level α = 0.1), and the degree of heterogeneity was quantitatively judged by combining with *I*^2^. If there is no statistical heterogeneity (*P* > .1, *I*^*2*^ < 50%) between the results of each study, the fixed-effects model is used; if there is statistical heterogeneity (*P* < .1, *I*^*2*^ > 50%) between the results of each study, then subgroup analysis is used. For the multi-arm experiment included in this meta-analysis, grouping and merging are adopted, the multi-arm experiment is converted to a two-arm experiment, and the mean standard deviation is combined as required.^[13]^

If the study includes outcome indicators ≥10, the funnel plot will be used to assess whether publication bias is included in the trial. If there are differences in symmetry or distribution, there will be publication bias or small sample effects.^[14]^

### 3.6 Sensitivity analysis

The purpose of sensitivity analysis is to reduce the heterogeneity of research. When the heterogeneity is high, sensitivity analysis should be carried out to exclude the research progress involved one by one, observe the changes in the heterogeneity results, and make the experimental results more reliable and stable.^[15]^

### 3.7 Subgroup analysis

Subgroup analysis can try to classify and compare the sources of different intervention objects to determine whether the patient chooses the following as the analysis elements in the subgroup analysis because the treatment effect is significant under what circumstances.

### 3.8 Ethics and dissemination

Since this is a protocol for systematic review and meta-analysis, all data in this study come from published studies and do not involve patients, so ethical approval is not required. The results of this research will be distributed to peer-reviewed journals and published in relevant conferences.

## 4 Discussion

Aromatherapy is an auxiliary treatment method, which takes various forms such as inhalation aromatherapy and aroma massage, and absorbs it into the body through the skin, respiratory system, or gastrointestinal system, thereby improving body function and balance of mind and body. The most commonly used method of aromatherapy is lavender essential oil alone or mixed with lavender essential oil and other essential oils for inhalation or massage. It is more commonly used in improving sleep, treating anxiety and depression, pain relief, and antiemetic.^[16]^ For its mechanism of action, such as linalool and linalyl acetate contained in lavender, it acts on the hypothalamus, pituitary, and olfactory nerves to trigger memory and produce emotional responses, while reducing sympathetic nerve activity, increasing parasympathetic nerve activity, and slowing heart rate and blood pressure, so as to relax the body and mind and improve sleep.

The research results of Lin et al^[17]^ showed that the subjects of intervention in the study included college students, professional women, postmenopausal women, and so on, indicating that aromatherapy is applicable to a wide range of people and can be applied to patients with sleep disorders and healthy people. For the intervention period of aromatherapy, the effect of intervention period of <4 weeks seems to be better, indicating that aromatherapy can be used as an important auxiliary means to improve sleep in the short term. However, since there are only 2 studies in the subgroup of <4 weeks, the reliability of this conclusion still needs more research and verification.^[18]^ For the intervention subjects, the patients showed better curative effect, which is closely related to the relatively high level of physical and psychological discomfort in the patients. In the intervention method, only inhalation of aromatherapy has a curative effect on sleep disorders, which may be due to the fact that the essential oils are closer to the olfactory system during inhalation and are easier to be absorbed.

However, the effectiveness of aromatherapy in the treatment of insomnia has not been scientifically and systematically evaluated. This study aims to evaluate the clinical efficacy of aromatherapy in the treatment of patients with insomnia. The conclusions of this study can provide evidence-based medicine recommendations for aromatherapy treatment.

## Author contributions

**Conceptualization:** Xin Song, Weiyu Jiang, Lisheng Jiang, Minghua Ye.

**Data curation:** Xin Song, Jiahua Peng, Minghua Ye, Lisheng Jiang.

**Formal analysis:** Xin Song, Weiyu Jiang.

**Project administration:** Xin Song, Weiyu Jiang, Minghua Ye.

**Supervision:** Jiahua Peng, Minghua Ye, Lisheng Jiang.

**Writing – review & editing:** Xin Song, Lisheng Jiang.
